# Development of a *Plasmodium berghei* transgenic parasite expressing the full-length *Plasmodium vivax* circumsporozoite VK247 protein for testing vaccine efficacy in a murine model

**DOI:** 10.1186/s12936-016-1297-3

**Published:** 2016-04-30

**Authors:** Masanori Mizutani, Shinya Fukumoto, Adam Patrice Soubeiga, Akira Soga, Mitsuhiro Iyori, Shigeto Yoshida

**Affiliations:** Laboratory of Vaccinology and Applied Immunology, Kanazawa University School of Pharmacy, Kanazawa, Japan; National Research Center for Protozoan Diseases, Obihiro University of Agriculture and Veterinary Medicine, Obihiro, Japan

**Keywords:** Circumsporozoite protein, *Plasmodiumberghei*, *Plasmodium vivax*, PvCSP, Transgenic parasite, VK210, VK247, Vaccine

## Abstract

**Background:**

The approach of using transgenic rodent malaria parasites to assess the immune system’s response to antigenic targets from a human malaria parasite has been shown to be useful for preclinical evaluation of new vaccine formulations. The transgenic *Plasmodium berghei* parasite line [PvCSP(VK210)/Pb] generated previously expresses the full-length circumsporozoite protein (CSP) VK210 from *Plasmodium vivax*. The transgenic parasite expresses one of the two most common alleles of CSP, defined by nine amino acids at the central repeat region of this protein. In the present study, a transgenic *P. berghei* parasite line [PvCSP(VK247)/Pb] expressing the full-length PvCSP(VK247), which is the alternative common allele, was generated and characterized.

**Methods:**

The *P. berghei* expressing full-length PvCSP(VK247) was generated and examined its applicability to CSP-based vaccine research by examining its biological characteristics in mosquitoes and mice.

**Results:**

Similar to PvCSP(VK210)/Pb, PvCSP(VK247)/Pb developed normally in mosquitoes and produced infectious sporozoites equipped to generate patent infections in mice. Invasion of HepG2 cells by PvCSP(VK247)/Pb sporozoites was inhibited by an anti-PvCSP(VK247) repeat monoclonal antibody (mAb), but not by an anti-PvCSP(VK210) repeat mAb.

**Conclusions:**

These two transgenic parasites thus far can be used to evaluate the potential efficacy of PvCSP-based vaccine candidates encompassing the two major genetic variants in preclinical trials.

## Background

Malaria is an ancient and life-threatening parasitic disease, which in 2015 was estimated to have caused 214 million cases and 438,000 deaths [1]. Of the five human malaria parasite species, *Plasmodium vivax* is currently the most widely distributed, with an “at-risk” population of almost three billion people (a third of the global population) [2, 3]. Although the importance of a vaccine against infection with *P. vivax* is recognized, the lack of a long-term in vitro culture system in red blood cells and suitable animal models of the disease as well as the complex life cycle of this parasite has hindered the development of a potent vaccine [4, 5].

The circumsporozoite protein (CSP), which covers the surface of *Plasmodium* sporozoites, underlies the most advanced protective malaria vaccine candidate to date [6, 7]. *Plasmodium vivax* CSP-derived subunit vaccine formulations have been shown to be safe, well tolerated, and immunogenic in malaria-naïve volunteers, and have, therefore, enabled progression of this vaccine candidate to protective efficacy trials [4, 8]. However, one complication impeding the development of a *P. vivax* vaccine is that, in contrast to *Plasmodium falciparum*, there are two common PvCSP alleles; these alleles consist of nine amino acids at the central repeat regions, GDRA[D/A]GQPA and ANGAGNQPG, thereby defining the sequence variants known as VK210 and VK247, respectively [9, 10]. Because VK210 and VK247 variants are both globally distributed [11–16], PvCSP-based vaccines need to induce protective immune responses to them both.

Recently, Espinosa et al. developed chimeric *Plasmodium berghei* parasites bearing the central repeat region of PvCSP(VK210) [17] and showed that antibodies against this central repeat region play an important role in protective immunity. The same authors speculated that full-length PvCSP vaccines would enhance the quality, magnitude, and breadth of protective antibody and T cell responses. More recently, a transgenic *P. berghei* parasite expressing the full-length PvCSP(VK210) allele [PvCSP(VK210)/Pb] has been developed and showed that the newly developed PvCSP vaccine elicited protective efficacy against infection with this transgenic parasite line [18].

In the study described here, a transgenic *P. berghei* parasite expressing the full-length PvCSP(VK247) allele [PvCSP(VK247)/Pb] was generated and characterized. The infectivity of the PvCSP(VK247)/Pb parasite line was comparable to that of the wild-type *P. berghei* ANKA 2.34 strain (WT-Pb) in vivo. PvCSP(VK247)/Pb sporozoites reacted strongly with an anti-PvCSP(VK247) mAb specific for the repeat region, and sporozoite invasion of HepG2 cells by PvCSP(VK247)/Pb was inhibited by the same mAb. These results suggest that these two transgenic parasites can be used to evaluate the potential efficacy of the two PvCSP-based vaccine candidates encompassing the two major genetic variants, making clinical trials of the two alternative forms of the CSP-based vaccine possible using a murine model of *P. vivax* infection.

## Methods

### Ethics statement

The experimental protocols used and all care and handling of the animals were in accordance with the guidelines for animal care and use prepared by Kanazawa University (Ref. no. 22118–1) and Obihiro University of Agriculture and Veterinary Medicine (Ref. no. 26–109).

### Cell lines and abs

HepG2 cells were maintained as described previously [19]. The monoclonal antibodies (mAbs) used were as follows: 2F2, mAb specific for the repeat sequence [DRA(D/A)GQPAG] of PvCSP(VK210) (MRA-184; Malaria Research and Reference Reagent Resource Center [MR4], Manassas, VA, USA); 2E10E9, mAb specific for the repeat sequence (ANGAGNQPG) of PvCSP(VK247) (MRA-185; MR4); 3D11, mAb specific for the repeat sequence of PbCSP of the ANKA strain (PbCSP) (MRA-100; MR4).

### Plasmid construction and parasite transfection

To construct the transfer plasmid for generating the PvCSP(VK247)/Pb transgenic parasite line in place of native PbCSP, the DNA sequence corresponding to amino acids His_24_-Asp_370_ of the entire PvCSP VK247 gene (GenBank accession number M69059) minus its signal peptide and glycosylphosphatidylinositol (GPI)-anchor sequence was amplified from pEU3-PvCSP(VK247) using pPvCSP-VK247-F4 and pPvCSP-VK247-R1 primers (Table 1). The PCR product was then cloned into pENTR (Invitrogen life Technologies, Carlsbad, CA, USA) to construct pENTR-PvCSP-VK247-*Mun*I. Our previous study confirmed that the GPI anchor of the PfCSP moieties derived from PvCSP(VK210) were able to function in the transgenic rodent parasites [18]. The DNA sequence corresponding to amino acids Lys_372_-Asn_395_ of the GPI anchor of PfCSP (Accession number AAN87615) was amplified from pBS-5′UTR-PfCSP-Tcell [20] using pPvCSP-VK247-F1 and pPfCSP-R7 primers (Table 1), and then cloned into pENTR to construct the pENTR-PvCSP-VK247-*Xma*I/*Mun*I plasmid. A 1.1-kb fragment of PvCSP(VK247) was excised from pENTR-PvCSP-VK247-*Mun*I by digestion with *Xma*I and *Mun*I and then inserted into the *Xma*I/*Mun*I sites of pENTR-PvCSP-VK247-*Xma*I/*Mun*I to construct pENTR-PvCSP-VK247-R1. A 1.7-kb fragment of the PvCSP(VK247) nucleotide sequence and GPI anchor nucleotide sequence of PfCSP was excised from pENTR-PvCSP-VK247-R1 by digestion with *Xma*I and *Sfu*I and then inserted into the *Xma*I/*Sfu*I sites of pBS-5′UTR-PvCSP(VK210)-dihydrofolate reductase (DHFR)-3′ [18] to construct pBS-5′UTR-PvCSP(VK247)-DHFR-3′-R1. The transgenic PvCSP(VK247)/Pb parasite was generated by transfection of WT-Pb with the linearized pBS-5′UTR-PvCSP(VK247)-DHFR-3′ plasmid, as described previously [21]. For genotype analysis, genomic PvCSP(VK247)/Pb DNA was extracted from PvCSP(VK247)/Pb-infected mouse blood using a QIAamp DNA Blood Mini kit (Qiagen, Hilden, Germany). The replacement of *Pbcsp* gene with the *Pvcsp* gene was confirmed by PCR using a set of forward and reverse gene-specific primers (Table 1).

**Table 1:** List of primers used in this study

Locus	Primer name	Sequence (5′–3′)	Purpose
PvCSP(VK247)	pPvCSP-VK247-F4	CACCCGGGCACAATGTAGATCTGTCCAAGGCCATA	Generation of PvCSP(VK247)/Pb
	pPvCSP-VK247-R1	CAATTGAACTATTTACGACATTAAACACACTGGAACACTTATCCATTGTACAAACATCAGTCTCAAGGTCATTCAA	
	pPvCSP-VK247-F1	CACCCGGGAATGTCGTAAATAGTTCAATTGGATTAATAATGGTA	
	pPfCSP-R7	GTCGACTGTTAAATGAACTTCGAAGAATTCATTTTTTGTG	
PbCSP	pPbCSP-F3	ATGAAGAAGTGTACCATTTTAGTTGTAGCG	Confirmation of integration by recombination for PvCSP genes
	pPbCSP-R3	TACAAATCCTAATGAATTGCTTACAATATT	
PvCSP(VK210)	pPvCSP-VK210-F4	CACCCGGGCACAATGTAGATCTGTCCAAGGCCATA	
	pPvCSP-VK210-R1	TCCATCTGCTCTGTCTCCTGGTTG	
PvCSP(VK247)	pPvCSP-VK247-F4	CACCCGGGCACAATGTAGATCTGTCCAAGGCCATA	
	pPvCSP-VK247-R3	ATTGCCAGCCCCATTTGCTCCTGGTTG	
Pb 18 s rRNA	p18 s rRNA-F1	AAGCATTAAATAAAGCGAATACATCCTTAC	Confirmation of integration by recombination for PvCSP genes and invasion assay
	p18 s rRNA-R1	GGAGATTGGTTTTGACGTTTATGTG	
Human action	pHA067803-F	TGGCACCCAGCACAATGAA	Invasion assay
	pHA067803-R	CTAAGTCATAGTCCGCCTAGAAGCA	

### Western blotting

Sporozoites were separated by 10 % sodium dodecyl sulfate–polyacrylamide gel electrophoresis, transferred to an Immobilon-FL PVDF membrane (Merck Millipore, Guyancourt, France) and then probed with either of the following mAbs: 3D11, 2F2, or 2E10E9. Blots probed with the appropriate secondary Abs conjugated to IRDye 800 (Rockland Immunochemicals, Gilbertsville, PA, USA) with the same membranes were visualized using an Odyssey infrared imager (LI-COR, Lincoln, NE, USA).

### Indirect immunofluorescence assay (IFA)

Sporozoites isolated from mosquito salivary glands were loaded onto glass slides and fixed as described previously [18]. Each slide was stained with an Alexa Fluor 488-conjugated 2E10E9 mAb and mounted with a drop of VECTASHIELD containing 4′, 6′-diamidino-2-phenylindole (Vector Laboratories, Burlingame, CA, USA). An LSM710 inverted laser scanning microscope (Carl Zeiss, Tokyo, Japan) was used for image acquisition.

### Infectivity and fitness of PvCSP(VK247)/Pb parasites

*Anopheles stephensi* mosquitoes (SDA 500 strain) were allowed to feed either on WT-Pb, PvCSP(VK210)/Pb, or PvCSP(VK247)/Pb parasite-infected mice. The infectivity of these parasites from mice to mosquitoes was assessed at day 14 post-feeding. Mosquito midguts were dissected in sterile PBS and stained with mercury chromate solution as described previously [21], after which the prevalence and numbers of oocysts were recorded. Sporozoite development in the mosquito salivary glands was assessed on day 21 after the blood meal was taken. Mice, bitten by three to seven mosquitoes infected with either PvCSP(VK210)/Pb or PvCSP(VK247)/Pb were checked for the development of blood-stage parasites by microscopic examination of Giemsa-stained thin blood smears.

### Inhibition of sporozoite infectivity in vitro

HepG2 cells were seeded at a density of 5 × 10^4^ cells/well in a collagen type I-coated 48-well plate 48 h prior to the addition of PvCSP(VK247)/Pb sporozoites. Sporozoites (2 × 10^3^), isolated from mosquito salivary glands and then incubated with 100-fold dilutions of 2F2 or 2E10E9 mAbs, were added to the HepG2 cells. The sporozoites were incubated with the cells for 72 h, the culture media was changed every 24 h, and total RNA was extracted from the cells using a QIAamp RNA blood mini kit (Qiagen). First-strand cDNA synthesis was carried out using MultiScribe™ reverse transcriptase (Applied Biosystems, Life Technologies, Foster City, CA, USA) with random primers. Semi-quantitative PCR was performed with *P. berghei* 18s rRNA and human β-actin primers (Table 1). *P. berghei* 18s rRNA copy numbers reflect the exoerythrocytic forms (EEF) numbers in the HepG2 cells [22].

### Statistical analyses

Statistical significance was assessed using the Kruskal–Wallis test to examine differences in oocyst or sporozoite counts per mosquito. Fisher’s exact probability test was used to examine differences in the infection prevalence rates (mosquitoes and mice). The Mann–Whitney test was used to examine differences in the parasitaemia of the mice. Values where *P* was <0.05 were considered statistically significant. Statistical analyses were performed using Prism version 6 (GraphPad Software Inc., La Jolla, CA, USA).

## Results

### Generation and biological characterization of PvCSP(VK247)/Pb parasites

**Fig. 1:**
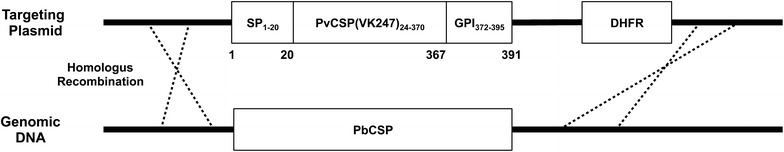
Schematic of the genomic region targeted by the PvCSP(VK247)/Pb cassette. The gene cassette consisted of the PbCSP signal sequence (SP) and the PvCSP(VK247) [PvCSP(VK247)_24–370_] genomic region fused to the region encoding the N-terminus of the glycosylphosphatidylinositol (GPI) anchor of PfCSP. The numbers indicate the amino acid positions of the SP, PvCSP(VK247), and GPI. SP: signal peptide of the PbCSP region corresponding to amino acids 1–20; PvCSP(VK247)_24–370_: PvCSP(VK247) region corresponding to amino acids 24–370; GPI: GPI anchor of the PfCSP region corresponding to amino acids 372–395. The PbCSP gene was replaced with the PvCSP(VK247) cassette and the dihydrofolate reductase (DHFR) selectable marker

Using a plasmid encoding the full-length *Pvcsp*(VK247) gene and the dihydrofolate reductase (DHFR) gene (which confers resistance to pyrimethamine) to transfect the parasites, the PvCSP(VK247)/Pb parasite was generated (Fig. 1). Briefly, the full-length *Pvcsp*(VK247) gene consists of the regions encoding the N-terminal signal peptide and C-terminal GPI anchor, both of which are derived from *Pfcsp*. The *P. vivax* replacement cassette represents the CSP sequence of the *P. vivax* VK247 strain, from amino acids 24–370. WT-Pb blood stage parasites were transfected in vitro with a plasmid linearized by the *Xho*I restriction enzyme, as described previously [21]. The transfected parasites, injected intravenously into BALB/c mice, were selected under pyrimethamine treatment. Ten days later, drug-resistant parasites were recovered and cloned by limiting dilution.

### Characterization of PvCSP(VK247)/Pb sporozoites with mAbs

**Fig. 2:**
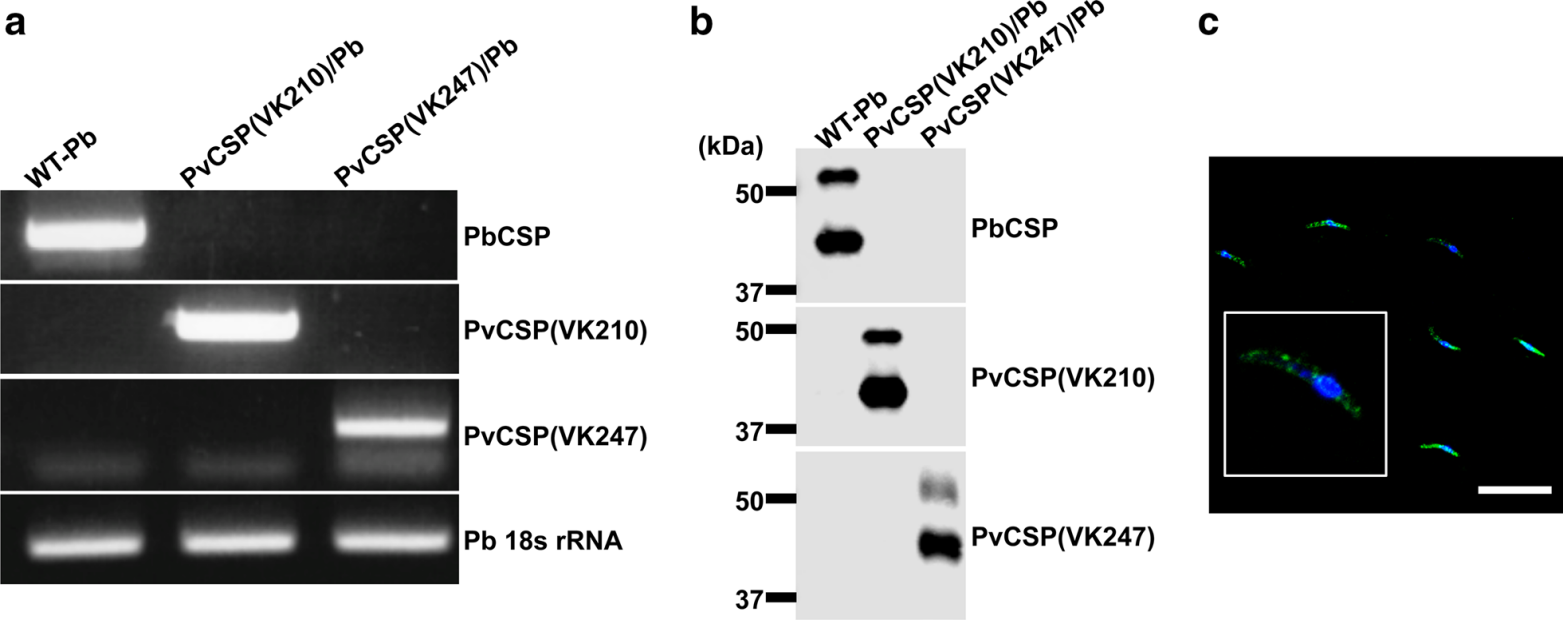
PvCSP(VK247) expression in PvCSP(VK247)/Pb sporozoites. **a** Replacement of the wild-type PbCSP gene with the PvCSP(VK210) gene or the PvCSP(VK247) gene in the PvCSP(VK210)/Pb or PvCSP(VK247)/Pb parasite lines was verified by PCR using genomic DNA from WT-Pb, PvCSP(VK210)/Pb, or PvCSP(VK247)/Pb, respectively. Pb 18s rRNA was used as a loading control. **b** The salivary glands from mosquitoes infected with the transgenic parasites were lysed and loaded onto 10 % gels, and then immunoblotted with the 3D11, 2F2, or 2E10E9 mAbs. **c** PvCSP(VK247)/Pb sporozoites were probed with the 2E10E9 mAb conjugated with Alexa Flour 488 (*green*). Parasite nuclei were visualized by DAPI (*blue*). *Bar* = 20 μm

Gene replacement by genetic recombination and genomic integration of the *Pvcsp*(VK247) gene cassette was confirmed in the transgenic parasite, PvCSP(VK247)/Pb (Fig. 2a). The western blotting data showed clearly that the PvCSP(VK247)/Pb sporozoites stained positive with the 2E10E9 mAb and negative with the 3D11 mAb or the 2F2 mAb (Fig. 2b). The relative molecular mass (*Mr*) of the PvCSP(VK247) protein is 37.0 kDa (Fig. 2b), a value lower than its predicted molecular weight (~50 kDa), and possibly resulting from cleavage of the precursor because cleavage of CSP occurs on the sporozoite surface when parasites contact hepatocytes [23, 24]. No cross-reactivity was observed between the 2E10E9 mAb and either WT-Pb, PvCSP(VK210)/Pb or PvCSP(VK247)/Pb sporozoites. IFAs showed that the PvCSP(VK247) protein was expressed on the surface of each sporozoite isolated from the salivary glands of PvCSP(VK247)/Pb-infected mosquitoes (Fig. 2c).

### Infectivity of PvCSP(VK247)/Pb parasites in mosquitoes and mice

**Fig. 3:**
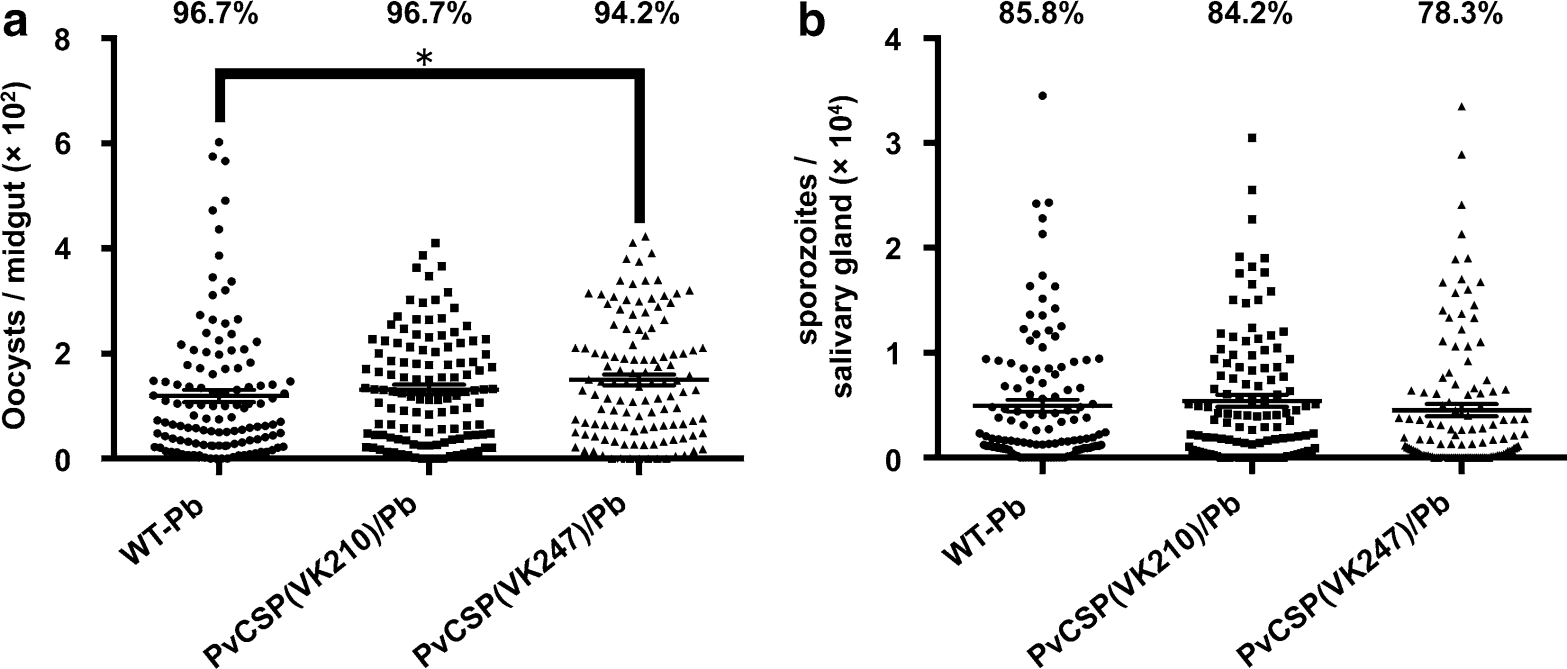
Developmental characteristics and the infectivity and fitness of profiles of PvCSP(VK247)/Pb parasites in *Anopheles stephensi* mosquitoes. The number of oocysts per mosquito and the percentage of infected mosquitoes were comparable among the WT-Pb (n = 120), PvCSP(VK210)/Pb (n = 120), and PvCSP(VK247)/Pb (n = 120) groups. **a** Midgut oocysts in the mosquitoes were examined 14 days after an infectious feed. Data are the mean number of oocysts observed (± SEM). **b** Salivary gland sporozoites in mosquitoes were examined 21 days after an infectious feed. Data are the mean number of sporozoites observed (± SEM). Percentages on* top* of the* panels* indicate the infectivity rates. Statistical significance was determined by the Kruskal–Wallis test (oocyst or sporozoite intensity) or Fisher’s exact probability test (oocyst or sporozoite prevalence). *P < 0.05

**Fig. 4:**
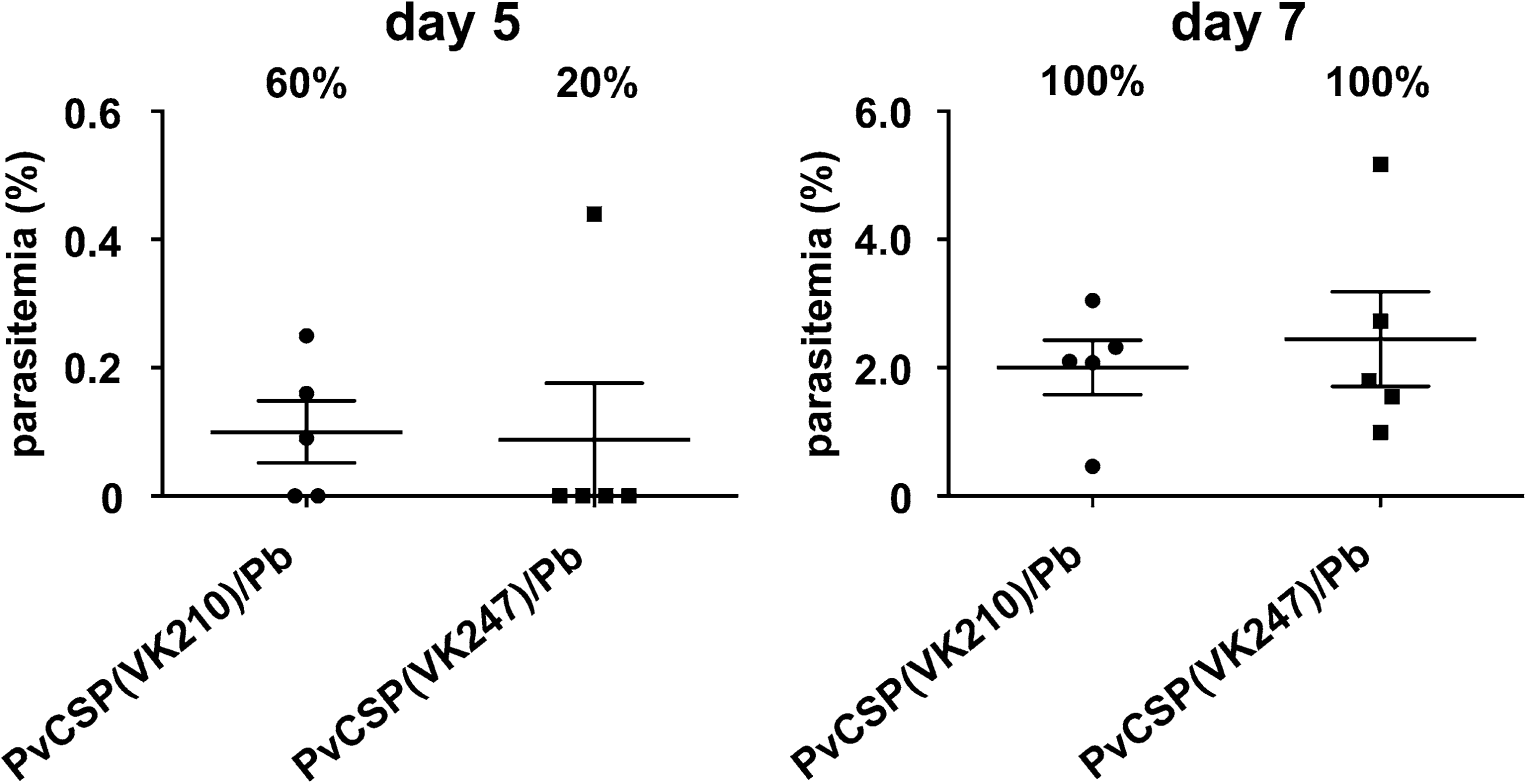
Parasitaemia in mice infected with PvCSP/Pb transgenic parasites after receiving infectious mosquito bites. Each mouse (five mice/group) was bitten by three to seven mosquitoes infected with PvCSP(VK210)/Pb or PvCSP(VK247)/Pb. Giemsa-stained thin smears of mouse tail blood were prepared on day 5 and 7 after the mice had received the mosquito bites. Data are the means (± SEM) of the results. The percentages on the* top* of the* panels* indicate the infectivity rates. Statistical significance was determined by the Mann–Whitney test (percentage parasitaemia) and Fisher’s exact probability test (infectivity rates)

Oocyst and sporozoite counts for the PvCSP(VK247)/Pb transgenic parasite were compared with those of the WT-Pb or the transgenic PvCSP(VK210)/Pb parasite lines. On day 14 post feeding, the PvCSP(VK247)/Pb-infected mosquito midguts contained approximately 150 healthy-looking oocysts per midgut. Oocyst intensity and prevalence in the PvCSP(VK247)/Pb line were not reduced compared with those of the WT-Pb or the PvCSP(VK210)/Pb parasite lines (Table 2; Fig. 3a). On day 21, after dissecting the mosquito salivary glands, the total number and percentage prevalence of sporozoites from PvCSP(VK247)/Pb were comparable to those of the WT-Pb and the PvCSP(VK247)/Pb lines (Table 2; Fig. 3b). To examine whether PvCSP(VK247)/Pb transgenic parasites had adapted to their in vivo environment, BALB/c mice were challenged through the biting of three to seven PvCSP(VK247)/Pb- or PvCSP(VK210)/Pb-infected mosquitoes. At 5 and 7 days post-infection, the parasite prevalence and parasitaemias of the subsequent blood-stage infections were monitored. Although the percentage of the infected mice differed between the PvCSP(VK210)/Pb and the PvCSP(VK247)/Pb lines on day 5, there was no statistically significant difference between them (Fig. 4). All the mice (7/7) became infected 7 days after being bitten by PvCSP(VK247)/Pb- or PvCSP(VK210)/Pb-infected mosquitoes. There was no significant difference between the parasitaemias attained by the mice from either parasite line on day 5 and 7. Thus, these results indicate that the PvCSP(VK247)/Pb and the PvCSP(VK210)/Pb lines were comparable in terms of their parasitaemias and infectivity profiles in mice. Figures 3 and 4 show that the PvCSP(VK247)/Pb parasite line behaved sufficiently similar to the WT-Pb or the PvCSP(VK210)/Pb lines in the mosquito and the mouse stages to allow it to be used for measuring the protective efficacy of CSP-based vaccines.

**Table 2:** Developmental characteristics and infectivity of PvCSP (VK247)/Pb parasites in *Anopheles stephensi* mosquitoes

Parasite	Oocysts per midgut (± SEM)	% of midgut infected	Sporozites per salivary gland (± SEM)	% of salivary gland infected
WT-Pb	119.49 (11.64)	96.7	4953 (565)	85.8
PvCSP(VK210)/Pb	131.91 (9.15)	96.7	5415 (555)	84.2
PvCSP(VK247)/Pb	150.14 (10.25)^a^	94.2	4533 (595)	78.3

Significance was assessed using the Kruskal–Wallis test (oocysts per midgut or sporozoites per salivary gland) or Fisher’s exact probability test (% of midguts or salivary glands infected parasites)

120 mosquitoes were examined in each experiment

^a^P < 0.05 (Kruskal–Wallis test) compared with the WT-Pb parasite

### The 2E10E9 mAb inhibits PvCSP(VK247)/Pb sporozoite invasion of HepG2 cells in vitro

**Fig. 5:**
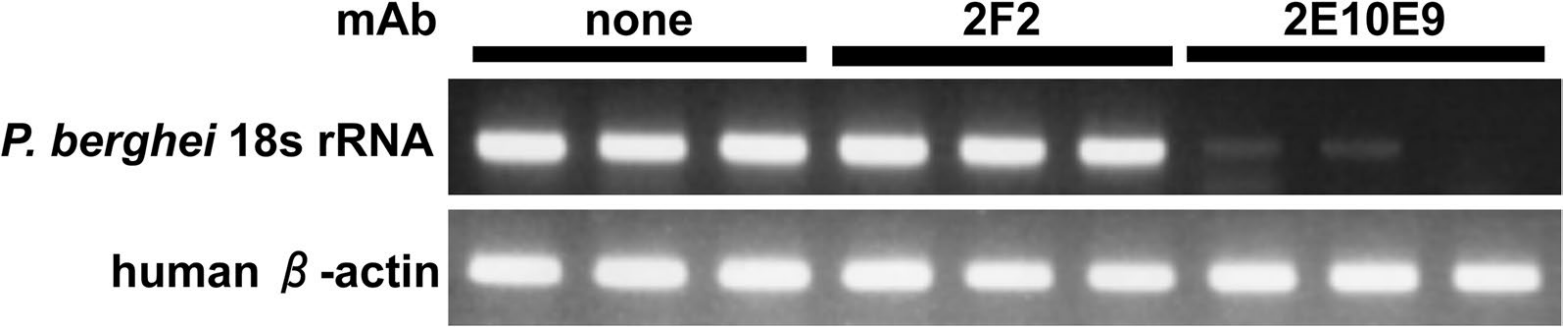
Sporozoite neutralization assay. PvCSP(VK247)/Pb sporozoites (2 × 10^3^) were incubated with antibodies for 40 min on ice and then added to cultured HepG2 cells. After 72 h, the medium was removed and total RNA was isolated. Reverse transcriptase-PCR was performed to detect *Plasmodium berghei* 18s rRNA, which is liver-stage specific, and human β-actin was used as a control. *Data* represent two independent biological replicates

To evaluate the utility of the transgenic PvCSP(VK247)/Pb parasite line as a tool for assessing the efficacy of future vaccines, the neutralization capabilities of anti-PvCSP mAbs in vitro were tested. Figure 5 shows that PvCSP(VK247)/Pb sporozoite invasion of HepG2 cells was inhibited by the 2E10E9 mAb, but not by the 2F2 mAb. This result indicates that PvCSP(VK247)/Pb sporozoite invasion of HepG2 cells is inhibited by the mAb targeting the PvCSP repeat region of VK247.

## Discussion

In the present study shown the successful development, characterization, and applicability to CSP-based vaccine research of *P. berghei* parasites expressing the full-length PvCSP(VK247). The infectivity levels of PvCSP(VK247)/Pb from mosquitoes to mice and vice versa were comparable to those of WT-Pb or PvCSP(VK210)/Pb parasite lines. PvCSP(VK247)/Pb sporozoite invasion of HepG2 cells was inhibited by 2E10E9, a mAb that specifically recognizes the repeat sequence (ANGAGNQPG) of PvCSP(VK247). These results suggest that the PvCSP(VK247)/Pb parasite line can be used in sporozoite challenge tests evaluating PvCSP-based vaccines.

Our previous report has shown that the PvCSP(VK210)/Pb parasite line is a useful tool for evaluating the protective efficacies of new PvCSP-based vaccines in a murine model of malarial disease [18]. PvCSP(VK247)/Pb sporozoite invasion of HepG2 cells was specifically inhibited by the 2E10E9 mAb, but not by the 2F2 mAb. Therefore, the PvCSP(VK247)/Pb and PvCSP(VK210) parasite lines can be used to evaluate the potential efficacies of PvCSP-based vaccine candidates encompassing the two major genetic variants prior to clinical trials.

Since there is no human malaria parasite-animal challenge model to test protective or transmission blocking efficacy of new vaccine formulations, a murine model employing transgenic parasites would closely mimic vaccination. Furthermore, the use of mice could be more informative than the in vitro sporozoite neutralization assay or membrane feeding assay, which are only an in vitro method for assessment of humoral immune responses and may not truly represent the in vivo cellular immune responses. Based on this concept, several transgenic lines {e.g., PfCSP [20, 25–28], PvCSP(VK210) [17, 18], Pfs25 [29, 30], Pvs25 [31, 32], PfMSP1_19_ [33, 34], and PvTRAP [35]} have been successfully established by our and other groups to evaluate malaria vaccine candidates in murine models. These transgenic lines have proven to be an important tool in preclinical optimization of malaria vaccines as well as in future testing of sera from people vaccinated with the vaccines, without requiring *P. falciparum* and *P. vivax* cultures in the vaccination field sites in areas of endemicity.

Hence, use both of the PvCSP(VK210)/Pb and PvCSP(VK247)/Pb should facilitate the appraisal of new vaccine candidates with universal coverage of the allelic variants of malaria antigens while continuing to provide a practical platform for evaluating CSP-based protective immune responses against *P. vivax*.

## Conclusions

The present study has been described the successful development of the transgenic *P. berghei* parasite expressing the full-length *Pvcsp*(VK247) in place of the natural *P. berghei* counterpart gene. The infectivity levels of PvCSP(VK210)/Pb and PvCSP(VK247)/Pb transgenic lines from mosquitoes to mice and vice versa were comparable to that of WT-Pb. The transgenic parasites expressing either of the two allelic versions of full-length PvCSP are powerful tools for evaluating the functional in vivo efficacy of PvCSP-based vaccine-induced immune responses and identifying protective epitopes in such vaccines.
